# Identification of ACSF gene family as therapeutic targets and immune-associated biomarkers in hepatocellular carcinoma

**DOI:** 10.18632/aging.204323

**Published:** 2022-10-04

**Authors:** Zijin Zhao, Miaomiao Liu, Zhijie Xu, Yuan Cai, Bi Peng, Qiuju Liang, Yuanliang Yan, Wei Liu, Fanhua Kang, Qingchun He, Qianhui Hong, Wenqin Zhang, Jianbo Li, Jinwu Peng, Shuangshuang Zeng

**Affiliations:** 1 Department of Neurosurgery, Xiangya Hospital, Central South University, Changsha, China; 2 Clinical Research Center for Skull Base Surgery and Neuro-Oncology in Hunan Province, Changsha, China; 3 Department of Nuclear Medicine (PET-CT Central), Xiangya Hospital, Central South University, Changsha, China; 4 Department of Pathology, Xiangya Hospital, Central South University, Changsha, China; 5 Department of Pathology, Xiangya Changde Hospital, Changde, Hunan, China; 6 National Clinical Research Center for Geriatric Disorders, Xiangya Hospital, Central South University, Changsha, China; 7 Department of Pharmacy, Xiangya Hospital, Central South University, Changsha, China; 8 Department of Emergency, Xiangya Hospital, Central South University, Changsha, Hunan, China

**Keywords:** hepatocellular carcinoma, ACSF gene family, expression profiles, prognosis, infiltrating immune cells

## Abstract

Acyl-CoA synthetases (ACSs) are responsible for acyl-CoA synthesis from nonpolar hydrophilic fatty acids and play a vital role in many metabolic processes. As a category of ACS isozymes, members of ACS family (AACS, ACSF2-3, AASDH) participate in lipid metabolism; however, their expression patterns, regulatory mechanisms and effects in hepatocellular carcinoma (HCC) are poorly understood. Here, through evaluating the expression profiles of ACSF gene family, we found that upregulated AACS might be more significant and valuable in development and progression of HCC. Consequently, the mRNA expression levels of AACS and ACSF2 was accordantly increased in HCC. Kaplan-Meier plotter revealed that HCC patients with high level of AACS were highly related to a shorter overall survival time and relapse-free survival. Genetic alterations using cBioPortal revealed that the alteration rate of AACS were 5%. We also found that the functions of ACSF gene family were linked to several cancer-associated pathways, including long-term potentiation, phospholipase D signaling pathway and purine metabolism. TIMER database indicated that the AACS and ACSF2 had a strong relationship with the infiltration of six types of immune cells (macrophages, neutrophils, CD8+ T-cells, B-cells, CD4+ T-cells and dendritic cells). Next, Diseasemeth database revealed that the global methylation levels of ACSF2 was higher in HCC patients. In conclusion, this study firstly demonstrated that Acyl-CoA synthesis gene family, in particular, AACS, could be associated with immune microenvironment, thereby influencing the development and prognosis of patients with HCC.

## INTRODUCTION

Hepatocellular carcinoma (HCC) is known as one of the most aggressive and frequent malignant diseases to bring about global attention, which is ranking as the third of mortality and the sixth of morbidity in all malignant neoplasms [1, 2]. The onset of HCC is usually undetectable and subtle bringing about developing into middle and advanced stage [3]. The therapeutic result of this malignant tumor is unsatisfied resulting in the 5 years survival rate is less than 19% [4]. Accordingly, proper clinical diagnosis and exploration of precise molecular markers are essential to improve the prognosis of HCC patients.

Fatty acids (FA) play a vital part in human organisms, releasing energy with effect from adenosine triphosphate (ATP), being a significant component of bio-membranes as same as taking pate in metabolic pathways such as beta-oxidation, hydrolysis, activation, esterification and synthesis [5, 6]. The activation of FAs into serious metabolic pathways through the formation of high-energy CoA intermediates by acyl-CoA synthetases (ACSs) enzymes is required for their synthesis and degradation [7]. ACSs, which are located in the endoplasmic reticulum, mitochondria, and microsomal membranes, are enzymes that convert nonpolar hydrophilic FA substrates into acyl-CoA [6, 8, 9]. On the basis of the substrate preference and sequence identity regarding the chain lengths of fatty acids, the human genome includes 26 ACS genes distributed into 6 distinct families: the ACS short-chain family (ACS), ACS medium-chain family (ACSM), ACS long-chain family (ACSL), ACS very long-chain family (ACSVL), ACS bubblegum family (ACSBG) and ACSF family (ACSF) [10, 11]. This division indicates the chain length of these genes preferred substrate. In mammals, previous research confirmed four distinct ACSF clades, including ACSF1(AACS), ACSF2, ACSF3, ACSF4(AASDH). As one of the novel ACSs isozymes, AACS has been participated in regulation of ketone body utilization on fatty acids and/or cholesterol biosynthesis in rats [12]. ACSF3 is a candidate gene for human metabolism-related disease [13]. Furthermore, Xin et al. indicated that ACSF2 gene may act as an original biomarker for estimating prognosis of breast cancer patients [14].

However, the detailed analysis of the ACSF gene family expression profiles and functions in cancer needs to be further confirmation. Along with the establishment of amounts of bioinformatics databases and the express development of second-generation gene sequencing technology, we could clarify the ACSF family clearer in HCC pathogenesis and treatment based on a extensive analysis of the ACSF family. In this study, we first conducted a detailed and integrated biological function of the ACSF family (including AACS ACSF2-3 and AASDH) in HCC. Additionally, on the strength of multiple public bioinformatics databases, we explored the potential of ACSF family as prognostic biomarkers and therapeutic targets. The purpose of this research is to attempt to select a more appropriate therapy and precise prediction for the long-term prognosis of HCC patients (Supplementary Table 1).

## RESULTS

### Aberrant expression of ACSF gene family in HCC patients

**Figure 1:**
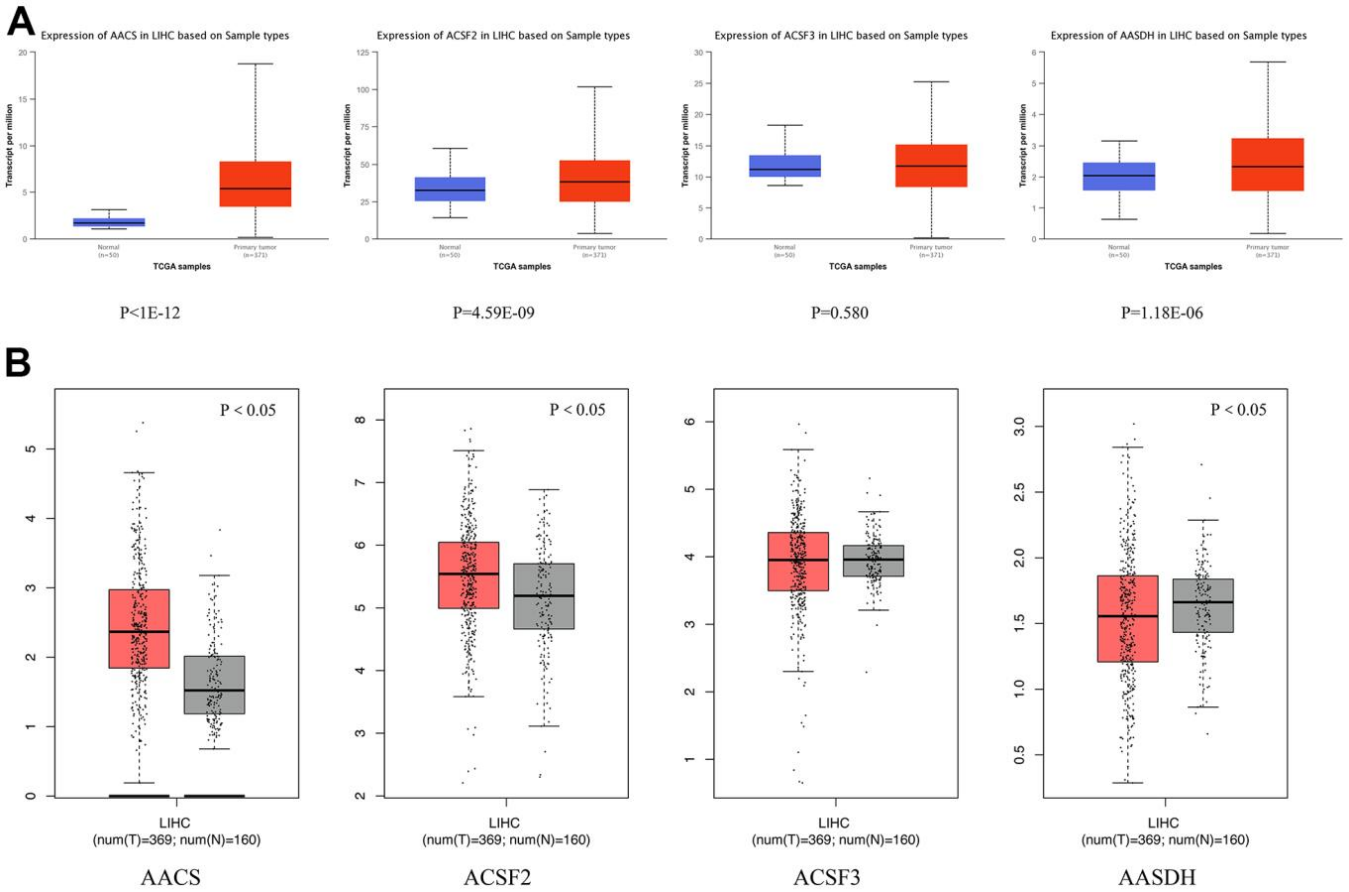
**Differential mRNA expression analysis of the ACSF gene family in HCC and normal tissues.** (**A**) The expression profiles were collected from the Ualcan databases. The red and blue graphs indicated the high-regulation and low-regulation, respectively. (**B**) The expression profiles were collected from the GEPIA databases. The red and gray graphs indicated the high-regulation and low-regulation, respectively.

We adopted ACSF gene both GEPIA and Ualcan database to evaluate the expression levels of this family members. The data revealed that all ACSF gene family members were significantly increased in most of the malignant neoplasms (Supplementary Figure 1). Then, we used Ualcan databases to detect the mRNA expression levels of ACSF gene family members in HCC tissues and adjacent normal tissues, as well as the levels of mRNA expression in HCC tissues and normal tissues were evaluated by the Wanderer database of ACSF gene family members. The results showed that the expression level of AACS (p <1E-12), ACSF2 (p = 4.59E-09) and AASDH (p = 1.18E-06) were significantly higher expressions in patients of HCC (Figure 1A). Then, the expression level of AACS (p < 0.05) and ACSF2 (p < 0.05) were remained significantly raised in HCC tissues according to GEPIA database, but the expression level of AASDH was reduced in tumor tissues than in normal tissues (Figure 1B). Moreover, we used the HCCDB database to analyze the expression levels of ACSF gene family members in HCC tissues and adjacent tissues. The analysis of data revealed that the expression of AACS (p = 7.850E-34), ACSF2 (p = 1.900E-10), and AASDH (p = 0.001596) in dataset of HCCDB4 and the expression of AACS (p = 5.510E-26), ACSF2 (p = 5.920E-14), and AASDH (p = 1.410E-11) in dataset of HCCDB18 in HCC tissues were both upregulated, separately (Table 1).

**Table 1:** The expression of ACSF gene family members in HCC tissues and adjacent tissues from the HCCDB database.

**Dataset**	**Gene**	**Type**	**Nums**	**Mean**	**STD**	**P-value**	**IQR**
HCCDB4	AACS	HCC	240	6.476	0.3388	7.850e-34	0.4992
		Adjacent	193	6.143	0.1577		0.2309
	ACSF2	HCC	240	7.877	0.5510	1.900e-10	0.7366
		Adjacent	193	7.601	0.3149		0.4096
	ACSF3	HCC	240	6.985	0.3381	0.2203	0.4525
		Adjacent	193	6.949	0.2843		0.3756
	AASDH	HCC	240	6.559	0.3469	0.001596	0.5615
		Adjacent	193	6.468	0.2476		0.3966
HCCDB18	AACS	HCC	212	1.893	0.7057	5.510e-26	0.8800
		Adjacent	177	1.237	0.4046		0.4600
	ACSF2	HCC	212	4.549	0.7931	5.920e-14	1.030
		Adjacent	177	4.035	0.4890		0.6200
	ACSF3	HCC	212	2.842	0.6466	0.9923	0.8525
		Adjacent	177	2.842	0.4040		0.5300
	AASDH	HCC	212	1.900	0.3491	1.410e-11	0.4425
		Adjacent	177	1.703	0.1963		0.2500

**Figure 2:**
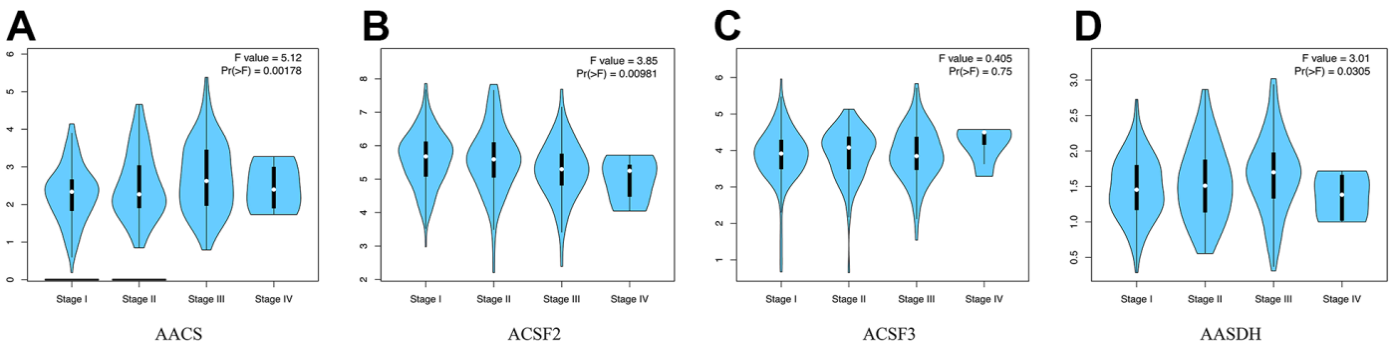
**The relationship between the expression of the ACSF gene family and the pathological stage of HCC patients (GEPIA).** (**A**–**D**) GEPIA databases were used to evaluate the correlations of the ACSF gene family with the pathological stage of HCC patients.

Then, the correlations between the pathological stages of patients with HCC and the expression profiles of ACSF gene family were analyzed. From Figure 2A–2D, the expression of AACS (p = 0.00178), ACSF1 (p = 0.00981) and AASDH (p = 0.0305) indicated a significant difference with four clinical stage subgroups. These results demonstrated that abnormal expression of ACSF gene family members might involve in tumor progression in patients with HCC.

### The prognostic value of the ACSF gene family in HCC patients

**Figure 3:**
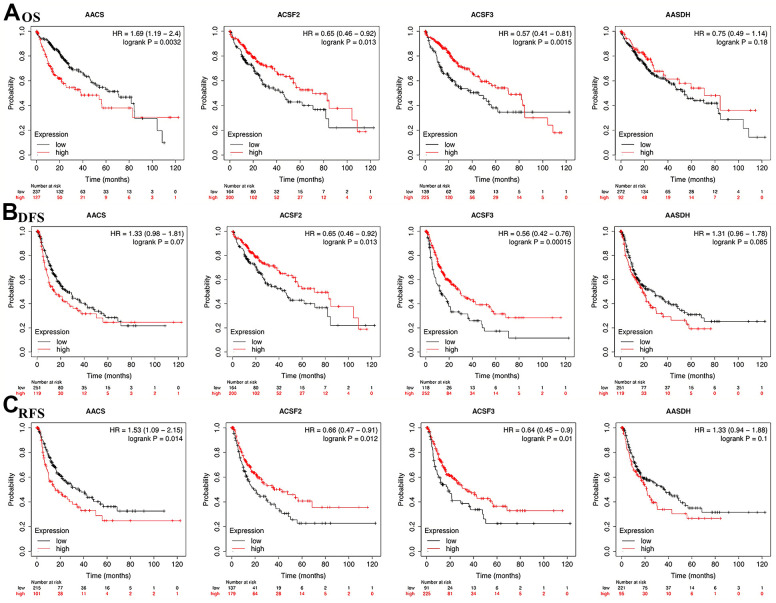
**The correlations of ACSF gene family expression with OS and PFS in HCC patients.** (**A**–**C**) Kaplan-Meier plotter was used to assess the correlation of ACSF gene family members with the patients’ OS (**A**), DFS (**B**) and RFS (**C**).

To explore the prognostic value of the ACSF gene family in patients with HCC, the association between the survival endpoints and expression of ACSF gene members, such as overall survival (OS), disease-free survival (DFS) and relapse-free survival (RFS) were further explored through Kaplan-Meier plotter. We found that HCC patients with depressed expression of AACS (p=0.0032) was correlated with a better OS, while elevated transcriptional level of ACSF2 (p=0.013) and ACSF3 (p=0.0015) were greatly related to a better OS time (Figure 3A). In the meantime, the prognostic significance of ACSF gene family on the DFS of HCC patients were evaluated. As shown in Figure 3B, we found that high levels of ACSF2 (p = 0.013) and ACSF3 (p = 0.00015) have an intimate correlation with a longer DFS time. Similarly, low expression of AACS (p=0.014), high expression of ACSF2 (p=0.012) and ACSF3 (p=0.01) that meaning a better RFS (Figure 3C).

### Genetic alteration and functional enrichment analyses of ACSF gene family in HCC patients

**Figure 4:**
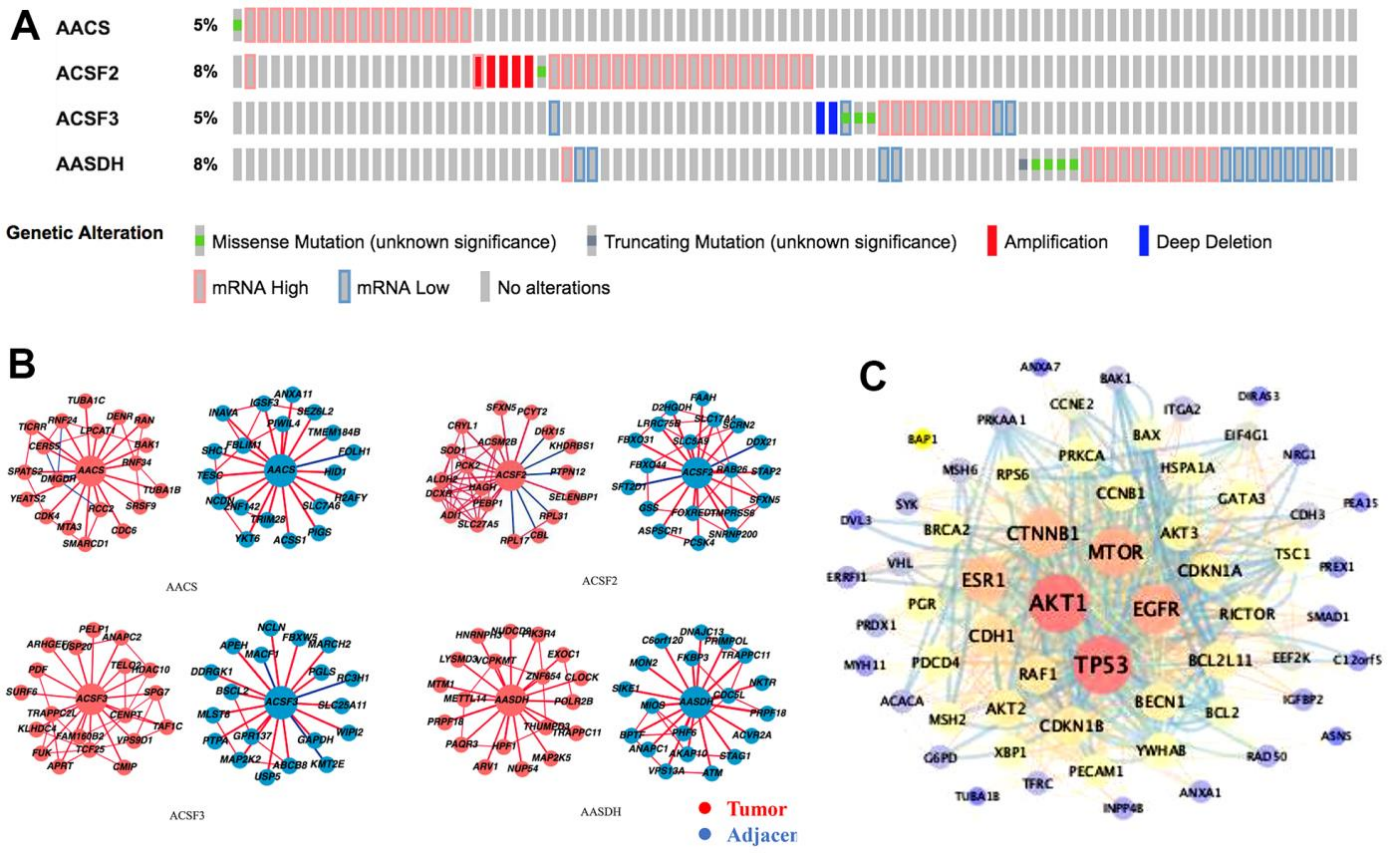
**Genetic alterations and interaction analyses of the ACSF gene family in HCC patients.** (**A**) Genetic alteration of the ACSF gene family in HCC patients analyzed with cBioPortal. (**B**) The HCCDB database was used to analyze the ACSF gene family co-expressed genes in HCC and adjacent tissue samples. (**C**) The most frequently altered genes identified from cBioPortal that are linked to the ACSF gene family in HCC patients.

In the ACSF gene family in LIHC, the frequency and types of alteration profiles were acquired through the cBioPortal tool and TCGA database. As shown in Figure 4A, the results showed that the alternation rate of ACSF2 and AASDH was the highest in 8% of cases, whereas the other family members are 5% (for AACS and ACSF3). In the matter of genetic alterations types, mRNA high alteration was the main genetic alterations in all ACSF gene family. However, in several HCC patients, gene amplification alteration was also observed in ACSF2, and mRNA low alteration were found in ACSF3 and AASDH (Figure 4A).

Secondly, the associated molecules of ACSF gene family were evaluated by using HCCDB database indicating that the positive molecules in all ACSF gene family had varied significantly between the adjacent normal tissues and neoplasm tissues, respectively (Figure 4B). Further, we abstracted 202 most frequently altered genes which were significantly associated with ACSF gene family in HCC from the cBioPortal tool (Supplementary Table 2). Then, the analysis results proved that many hub genes, such as AKT1, TP53, MTOR, MAPK1, EGFR and CTNNB1, were closely participated in the biological behavior prognosis in ACSF gene family (Figure 4C). These results demonstrated that the ACSF gene family involved in the adjustment of varieties of signaling pathways influenced the LIHC pathophysiology.

**Figure 5:**
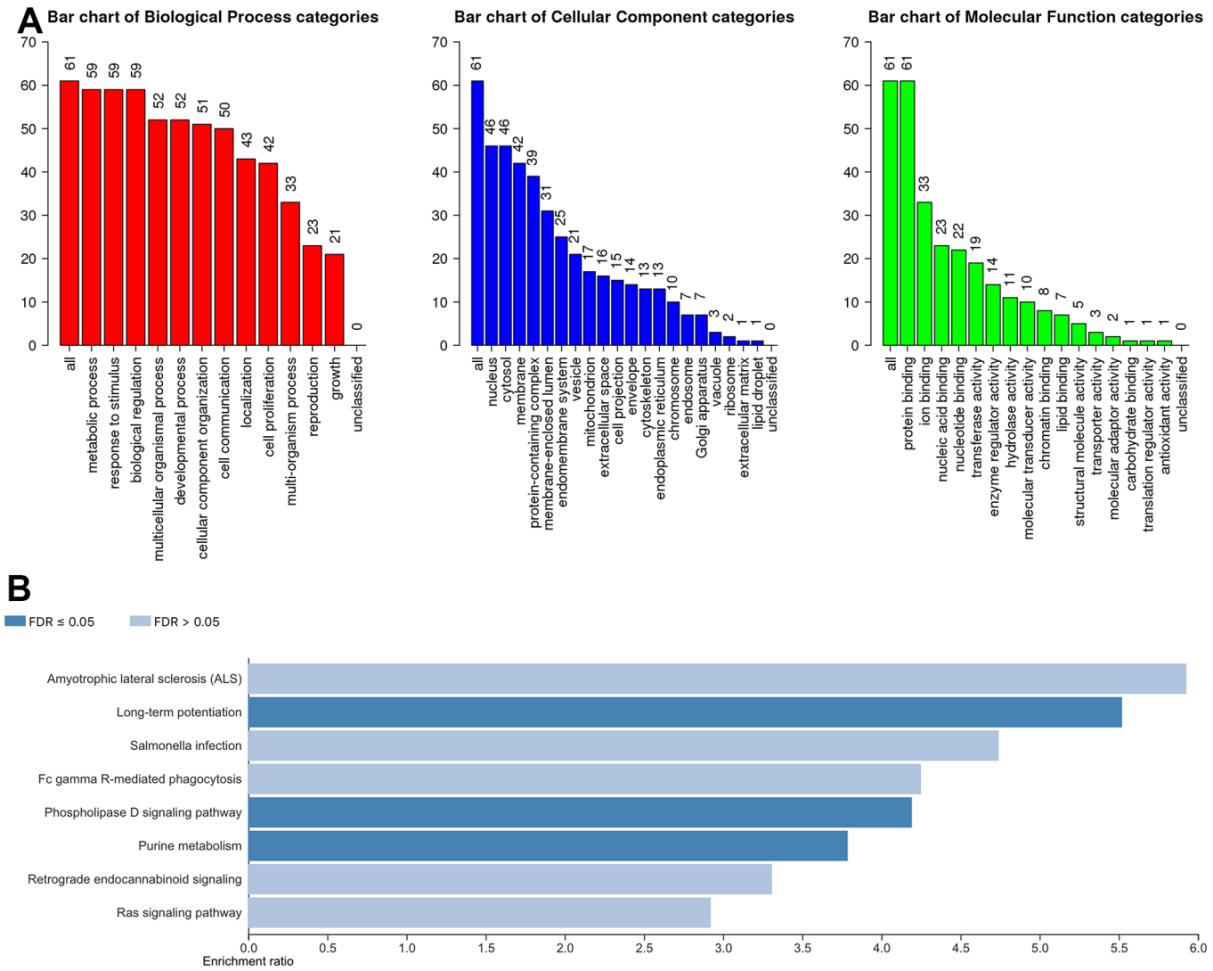
**The biological pathways of the ACSF gene family were evaluated by the WebGestalt database.** (**A**) Bar plot of GO enrichment in cellular components, biological processes, and molecular functions. (**B**) The bar plot of KEGG enrichment.

To further understand the potential biological functions of ACSF gene family, on the basis of the 202 ACSF-associated genes, we selected the WebGestalt database to perform the functional enrichment analysis. Accordingly, ACSF gene family members were most highly concentrated in biological function (BF) category like metabolic process, biological regulation, response to stimulus, developmental process and multicellular organismal process. In the aspect of categories of cellular component (CC)cytosol, nucleus, membrane, protein-containing complex and membrane-enclosed lumen were extremely enriched. Moreover, the ACSF gene family members were mainly enriched in the following categories of molecular function (MF), including transferase activity, ion binding, nucleic acid binding, nucleotide binding and protein binding (Figure 5A). Simultaneously, the Kyoto Encyclopedia of Genes and Genomes (KEGG) pathway analysis was implemented to suggest that the enriched pathways were mainly involved in results displayed in Figure 5B. Obviously, some cancer-related pathways, including long-term potentiation, phospholipase D signaling pathway and purine metabolism, which we can demonstrate, were significantly related to the feasible biological functions of ACSF gene family involved in the occurrence and development of HCC.

### Correlation between immune cell infiltration and each ACSF gene family

**Figure 6:**
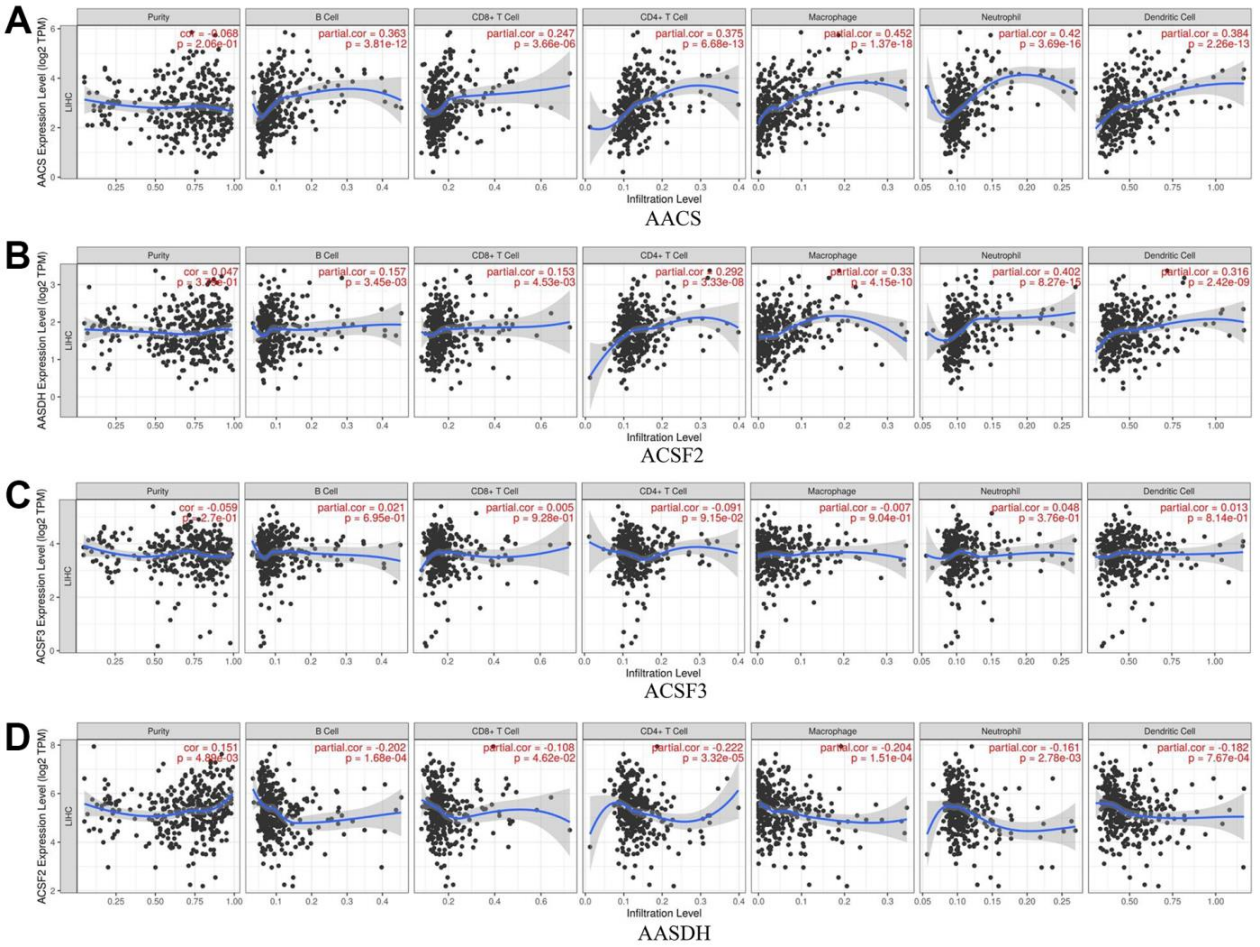
**The relationship between immune cell infiltration and the expression of the ACSF gene family.** (**A**–**D**) The Timer database was used to analyze the effect of (**A**) AACS, (**B**) ACSF2, (**C**) ACSF3, (**D**) AASDH on the abundance of immune cells in HCC patients.

The relationship between immune cell infiltration and each ACSF gene family was explored through the TIMER database. The AACS expression was affirmatively associated with the infiltration of B cells (Cor = 0.363, p = 3.81e-12), CD8+ T cells (Cor = 0.247, p = 3.66e-06), CD4+ T cells (Cor = 0.375, p = 6.68e-13), macrophages (Cor = 0.452, p = 1.37e-18), neutrophils (Cor =0.42, p = 3.69e-16) and dendritic cells (Cor =0.384, p = 2.26e-13) (Figure 6A). Meanwhile, the expression of ACSF2 was positively correlated with the infiltration of B cells (Cor = 0.157, p = 3.45e-03), CD8+ T cells (Cor = 0.153, p = 4.53e-03), CD4+ T cells (Cor = 0.292, p = 3.33e-08), macrophages (Cor = 0.33, p = 4.15e-10), neutrophils (Cor =0.402, p = 8.27e-15) and dendritic cells (Cor =0.316, p = 2.42e-09) (Figure 6B). Conversely, negative correlations between AASDH and the infiltration of B cells (Cor = -0.202, p = 1.68e-04), CD8+ T cells (Cor = -0.108, p = 4.62e-02), CD4+ T cells (Cor =- 0.222, p = 3.32e-05), macrophages (Cor = -0.204, p = 1.51e-04), neutrophils (Cor =-0.161, p = 2.78e-03) and dendritic cells (Cor =-0.182, p = 7.67e-04) were showed in Figure 6D. Nevertheless, the relationship between ACSF3 and immune cells was uncorrelated (Figure 6C). Surprisingly, above all six types of immune cells which were analyzed were positively linked to AACS and ACSF2. Furthermore, the Cox proportional hazard model proved that B cells (p = 0.016), CD8+ T cells (p = 0.019), macrophage cells (p = 0.032), dendritic cells (p = 0.001) and AACS (p = 0.014) correlated with the prognosis of HCC patient significantly (Table 2).

**Table 2:** The Cox proportional hazard model of ACSF domain family and seven types of immune cells in HCC patients from timer database.

	**Coef**	**HR**	**95%CI_l**	**95%CI_u**	**P.value**	**Sig**
Purity	1.154	3.170	1.079	9.318	0.036	*
B_cell	-8.868	0.000	0.000	0.186	0.016	*
CD8_Tcell	-5.989	0.003	0.000	0.373	0.019	*
CD4_Tcell	-6.418	0.002	0.000	1.389	0.062	
Macrophage	5.610	273.246	1.605	46529.528	0.032	*
Neutrophil	0.626	1.870	0.000	255364.856	0.917	
Dendritic	6.102	446.559	13.316	14975.279	0.001	**
AACS	0.300	1.349	1.063	1.713	0.014	*
ACSF2	0.022	1.023	0.838	1.247	0.825	
ACSF3	-0.228	0.796	0.608	1.042	0.097	**
AASDH	-0.115	0.892	0.598	1.329	0.573	

### Methylation level of ACSF gene family in HCC patients

**Figure 7:**
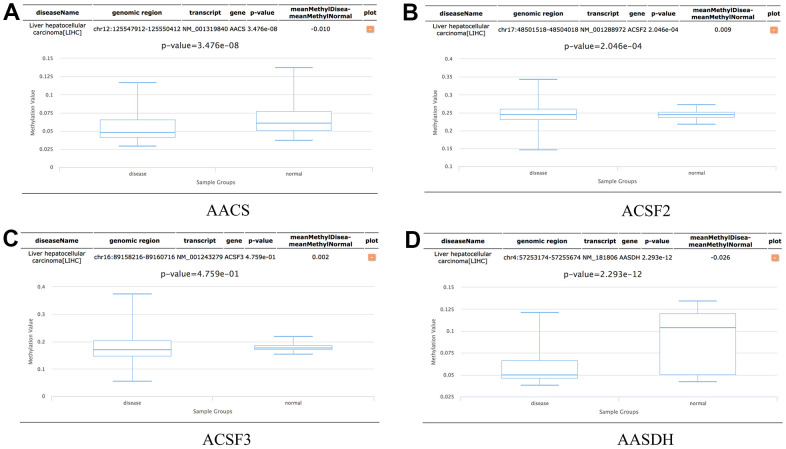
**The methylation levels of the ACSF gene family in HCC tissues.** (**A**–**D**) The methylation values of (**A**) AACS, (**B**) ACSF2, (**C**) ACSF3, (**D**) AASDH were evaluated using the Diseasemeth database.

In addition, the methylation levels of ACSF gene family in HCC patients were analyzed through the Diseasemeth database. The analysis data showed that the DNA methylation levels of AACS (p=3.476E-08) and AASDH (p=2.293E-12) were lower in HCC tissues than in normal tissues, but ACSF2 (p=2.046E-04) was higher in HCC than in normal cases (Figure 7A–7D). These DNA methylation changes could indicate possibly the difference of ACSF gene family in expression levels to a certain extent in HCC patients.

## DISCUSSION

The ACSF gene family catalyzes the fundamental, initial reaction in fatty acid metabolism of mammals [10]. Whereas, for the moment, little knowledge of ACSF family in cancer has been reported until now. Hasegawa et al. [15, 16] demonstrate that the limited proteolysis of AACS can encode the ketone body-utilizing enzyme, and is adjusted transcriptionally by SREBP-2. Meanwhile, the ketone body metabolism regulated by AACS plays a significant role in cholesterol metabolism. In literature, the data reveals that the ketone body is an fundamental element of cellular function and lipid metabolism, and exploration of transcriptional regulation of AACS could facilitate to clarify the role of ketone body utilization in neurological disorders [17]. To explain the possible mechanism of regulating the gene expression of human AACS and to elucidate the biological characteristics, Francesca et al. [18] isolated that the human promoter is a PPARγ (peroxisome-proliferator-activated receptor γ) target gene and PPARγ is employed to AACS promoter through the direct interaction with Sp1 (stimulating protein-1). Liu et al. [19] initially presented that the AACS may serve as new biomarker for a better recognition to the development, progression, and recurrence of GBM. Acyl-CoA synthetase family member 3 (ACSF3) is verified as the premier enzyme in the mtFAS pathway. The study found that deletion of ACSF3 enzymes could lead to combined malonic and methylmalonic aciduria (CMAMMA) [13]. Recent report emphasized that pharmacological activation of the SIRT3/ACSF3 pathway was an effective approach to alleviate NAFLD and protocatechuic acid was a novel candidate therapy for non-alcoholic fatty liver disease [20]. In addition, high expression of circ-AASDH was found to participate in the tumor size, clinical stage and regulate the expression level of E2F7 by sponging miR-140-3p, which involves in the malignant progression of LUAD [21].

According to our understanding, there is no systematic analysis aiming at the particular possibly functions and mechanisms of ACSF gene family in HCC. In this study, on the basis of the Ualcan, GEPIA, Kaplan-Meier plotter and HCCDB database, we demonstrated that the ACSF gene family expression levels are different between HCC tissues and normal cases. Our study explored the expression levels and function profiles of ACSF gene family in cancer for the first time. The data displayed that the AACS, ACSF2 and AASDH were strongly upregulated in HCC tissues and cells, meaning the potential to be oncogenes. Moreover, we implemented the relationship between ACSF family expression levels and HCC pathological stage. We detected that the expression level of AACS, ACSF2 and AASDH were raised positively with HCC stage progressed, indicating that these three proteins might involve in HCC progression. Additionally, only AACS was found to possess the prognostic value for HCC patients since the patients with high expression of AACS had a shorter OS and RFS time, while patients with high expression of ACSF2 and ACSF3 had a higher OS, DFS and RFS value in LIHC. These databases imply that AACS might be more valuable to be a crucial biomarker and therapeutic target for the patients with HCC. While, more specific evidence of studies and systemic elucidation of the mechanism of ACSF gene family in HCC needs to be further studied.

In addition, to better realize the potential mechanism of ACSF gene family in HCC, we used the GO and KEGG pathway enrichment analysis to examine the possible function of ACSF gene family. In HCC, the functional enrichment analysis in HCC proved that the amyotrophic lateral sclerosis (ALS), long-term potentiation and salmonella infection were the most relevant pathways in which ACSF family were involved. As reported by previous studies, serine biosynthesis pathway and tyrosine relative kinase inhibitors play critical roles in the regulation of HCC progression and immunotherapy [22, 23]. These findings suggest that ACSF gene family might participate in HCC progression through regulating signaling pathways.

Immune cell infiltration has been acknowledged as a critical role which is highly lined to the clinical outcomes, tumor responses and efficacy of immunotherapy [24–26]. A large amount of researches have concentrated on the correlation between tumors and immune cells [27, 28] and testified that immune cell infiltration could play important roles on the cancer immunotherapy and clinical outcomes [29, 30]. In this study, we found the expression of AACS and ACSF2 were greatly corelated with the infiltration of immune cells, including B cells, CD8+ T cells, CD4+ T cells, macrophages, neutrophils and dendritic cells, however, there was a negative correlation between AASDH and immune cell infiltration. These results highlighted that the ACSF gene family might regulate the immune cell infiltration, which could serve as the crucial indicators for the clinical outcomes of HCC patients.

To our knowledge, as an epigenetic modification, DNA methylation could regulate gene transcription and maintains genome stability. Meanwhile, methylation-associated gene silencing, which is a key component of post-transcriptional modification, plays an important role in tumor progression [31, 32]. In this study, the data revealed that the DNA methylation level of the AACS and AASDH gene levels are significantly reduced in HCC tissues, while the ACSF2 was upregulated, indicating the potential impact on the DNA methylation in the ACSF gene family.

In our study, some limitations can not be neglected. First, all of data and information we studied were mostly obtained from the bioinformatics databases. Consequently, additional and deep *in vitro* and *in vivo* experiments are needed to prove the functions and mechanisms of ACSF gene family in HCC. Next, in this study, the follow-up study of HCC patients was not performed concerning the therapeutic outcome. Therefore, the exploration of prognostic effects of the ACSF gene family on HCC patients is greatly imperative to strengthen the application values in clinical therapeutics.

To sum up, we comprehensively and systematically analyzed the molecular profiles of ACSF gene family in HCC from the aspect of bioinformatics, containing the gene expression levels, prognostic values and immune response. The results demonstrated that the ACSF gene family, particularly AACS, dominated enormous potential to be potential prognostic biomarkers and essential therapeutic targets in HCC. Therefore, our achievements might benefit of establishing more precise therapeutic strategies to modify the clinical outcomes and prognosis of HCC patients.

## MATERIALS AND METHODS

### Data acquisition

### GEPIA and Ualcan

Both of the Gene Expression Profiling Interactive Analysis (GEPIA) and Ualcan are databases which could support comprehensive expression analyses based on TCGA and GTEx data [33, 34]. We used these two databases to estimate the expression profiles of ACSF gene family in tumor and normal tissues. The p-value was a cutoff of 0.05.

### HCCDB

HCCDB could provide the visualization to the results of several computational analyses, containing differential expression level analysis, tumor-specific and tissue-specific expression analysis, survival analysis, and co-expression analysis [35]. In our study, we selected HCCDB4, HCCDB18 to identify whether there existed significant difference of protein expression between tumor tissues and adjacent tissues of ACSF gene family in HCC. Meanwhile, we showed co-expressed genes of ACSF gene family in HCC and adjacent tissues, respectively.

### Kaplan-Meier plotter

Kaplan-Meier plotter is a database exploring the correlation between gene expression with the prognostic role of multiple cancer patients [36]. In this study, we appraised the prognosis of patients with HCC through means of overall survival (OS) and progression free survival (PFS) curves. The data showed statistical significance if the p-value was a cutoff of 0.05.

### cBioPortal

cBioPortal provides analysis of genomic alteration data on more than 200 cancer patients [37]. In this study, the genetic alterations and co-expression profiles of ACSF gene family in HCC tissues were analyzed by searching cBioPortal.

### STRING

STRING is performed to evaluate potential protein-protein interactions (PPIs), and a database supporting an interactive network among numerous proteins [38]. Simultaneously, the STRING and Cytoscape were applied to analyze the ACSF gene family member-associated PPI network [39].

### GeneMANIA

GeneMANIA provides a convenient approach to identify protein-protein interactive networks [40]. We successfully identified the ACSF gene family associated genes by using the GeneMANIA.

### WebGestalt

WebGestalt is applied to provide a better understanding of gene interpretation, which can support enrichment results from this database [41]. In our study, we implemented Genes and Genomes (KEGG) pathway and Gene Ontology (GO) enrichment analysis linked to the ACSF gene family in HCC.

### TIMER

TIMER is a public resource employed to evaluate infiltration of immune cells in various types of cancer, providing more authoritative programs for an modified therapeutic response and prognosis [42]. Here, we primarily implemented correlation analysis between the expression of ACSF gene family with different types of immune cells.

### DiseaseMeth2.0

DiseaseMeth2.0 is a professional online database supporting DNA methylation information in various kinds of human diseases [43]. In our study, we explored the correlation between the ACSF gene family expression and DNA methylation levels. The p-value was a cutoff of 0.05.

## Supplementary Material

Supplementary Figure 1

Supplementary Table 1

Supplementary Table 2
