# Supplementary material for: Identification of ACSF gene family as therapeutic targets and immune-associated biomarkers in hepatocellular carcinoma
Source: Aging (Albany NY). 2022 Oct 4;14(19):7926–40. doi: 10.18632/aging.204323 (PMC9596203; doi:10.18632/aging.204323)
Supplement: Supplementary Figure 1 [file aging-14-204323-s001.pdf]

SUPPLEMENTARY FIGURE

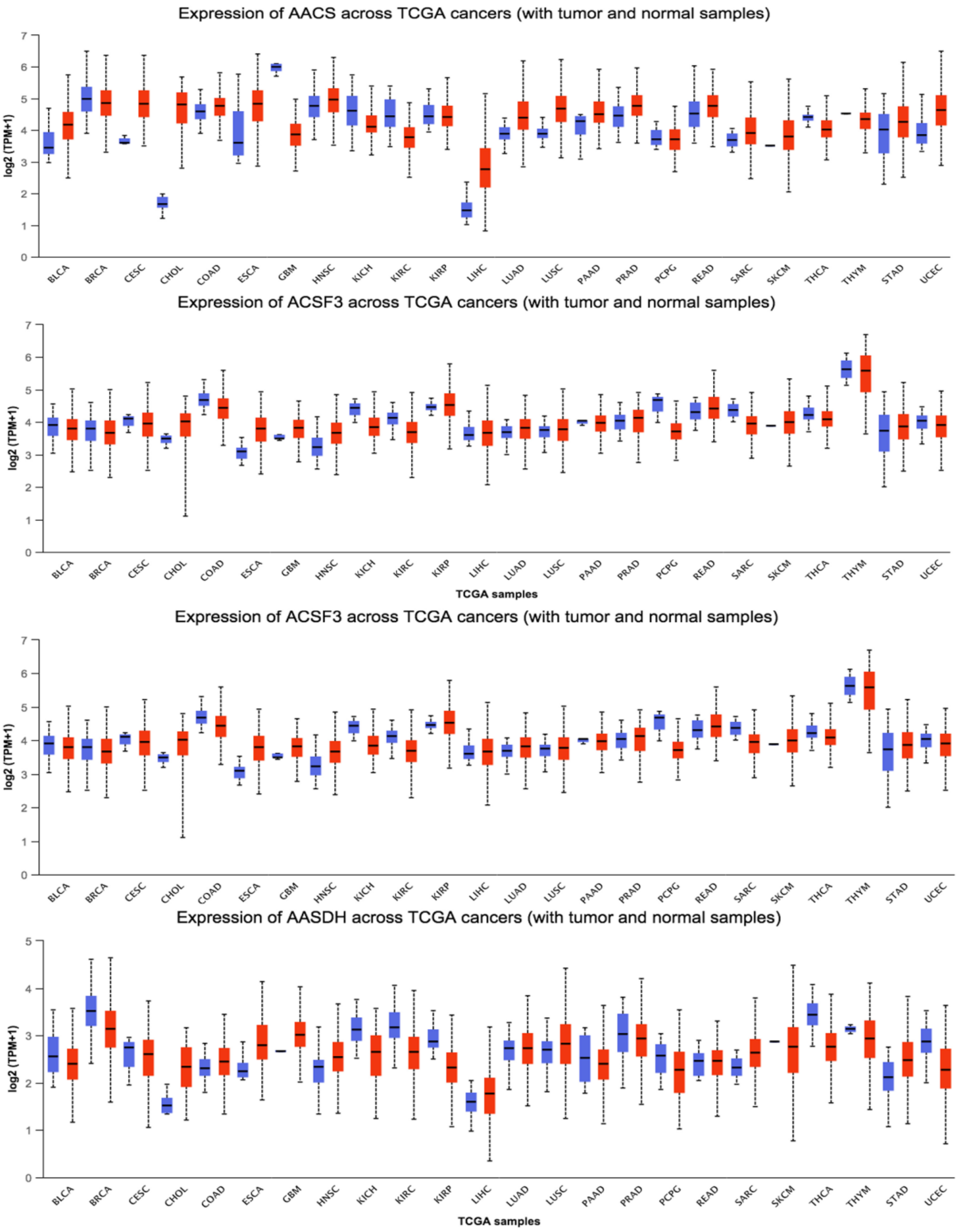

Supplementary Figure 1. ACSF gene family expression in different cancer types via the tumor immune estimation resource (Ualcan) database.
