# Supplementary material for: Identification of ACSF gene family as therapeutic targets and immune-associated biomarkers in hepatocellular carcinoma
Source: Aging (Albany NY). 2022 Oct 4;14(19):7926–40. doi: 10.18632/aging.204323 (PMC9596203; doi:10.18632/aging.204323)
Supplement: Supplementary Table 1 [file aging-14-204323-s002.pdf]

SUPPLEMENTARY TABLE

Supplementary Table 1. The main databases applied to evaluate the expression and functions of ACSF gene family in the biological process of HCC.

Databases	Authors	Publication date	Samples	URL
Ualcan	Chandrashekar DS. et al.	2017	Tissues	http://ualcan.path.uab.edu
GEPIA2	Tang Z. et al.	2019	Tissues	http://gepia.cancer-pku.cn/
HCCDB	Lian Q. et al.	2018	Tissues	http://lifeome.net/database/hccdb
Kaplan-Meierplotter	Gyorffy B. et al.	2005	Tissues	http://kmplot.com/analysis/
cBioPortal	Cerami E. et al.	2012	Tissues	http://www.cbioportal.org/
STRING v11	Szklarczyk D. et al.	2019	-	https://string-db.org/
GeneMANIA	Warde-Farley D. et al.	2010	-	http://genemania.org/
WebGestalt	Liao Y. et al.	2019	-	http://webgestalt.org/
Timer 2.0	Li T. et al.	2020	Tissues	https://cistrome.shinyapps.io/timer/
DiseaseMeth 2.0	Xiong Y. et al.	2017	Tissues	http://biobigdata.hrbmu.edu.cn/diseasemeth/

GEPIA, Gene expression profiling interactive analysis; WebGestalt, the web-based GEne SeT AnaLysis Toolkit.
