# Supplementary material for: Identification of ACSF gene family as therapeutic targets and immune-associated biomarkers in hepatocellular carcinoma
Source: Aging (Albany NY). 2022 Oct 4;14(19):7926–40. doi: 10.18632/aging.204323 (PMC9596203; doi:10.18632/aging.204323)
Supplement: Supplementary Table 2 [file aging-14-204323-s003.docx]

**Supplementary Table 2. The database of cBioportal applied to search the altered genes of the ACSF domain family in tissues of HCC.**

| **Gene** | **Cytoband** | **Log Ratio** | **p-Value** | **q-Value** | **expression** |
| --- | --- | --- | --- | --- | --- |
| IGFBP2 | 2q35 | -0.62 | 1.12E-03 | 0.226 | Unaltered group |
| ANXA1 | 9q21.13 | -0.31 | 0.0116 | 0.889 | Unaltered group |
| YWHAB | 20q13.12 | -0.05 | 0.0269 | 0.889 | Unaltered group |
| ASNS | 7q21.3 | -0.16 | 0.0476 | 0.889 | Unaltered group |
| NRAS | 1p13.2 | -0.04 | 0.0492 | 0.889 | Unaltered group |
| YWHAE | 17p13.3 | -0.04 | 0.0666 | 0.889 | Unaltered group |
| PRKCA | 17q24.2 | 0.14 | 0.0689 | 0.889 | Altered group |
| MYH9_PS1943 |  | -0.19 | 0.0717 | 0.889 | Unaltered group |
| MYH11 | 16p13.11 | -0.67 | 0.0723 | 0.889 | Unaltered group |
| PRKCA_PS657 |  | 0.13 | 0.0729 | 0.889 | Altered group |
| PGR | 11q22.1 | -0.07 | 0.0752 | 0.889 | Unaltered group |
| MTOR | 1p36.22 | 0.1 | 0.0762 | 0.889 | Altered group |
| ANXA7 | 10q22.2 | -0.05 | 0.0902 | 0.889 | Unaltered group |
| TIGAR | 12p13.32 | 0.28 | 0.1 | 0.889 | Altered group |
| HSPA1A | 6p21.33 | -0.14 | 0.102 | 0.889 | Unaltered group |
| ERRFI1 | 1p36.23 | -0.09 | 0.11 | 0.889 | Unaltered group |
| CCNE2 | 8q22.1 | 0.09 | 0.11 | 0.889 | Altered group |
| BCL2 | 18q21.33 | -0.08 | 0.118 | 0.889 | Unaltered group |
| RAF1 | 3p25.2 | 0.06 | 0.126 | 0.889 | Altered group |
| SMAD1 | 4q31.21 | 0.14 | 0.127 | 0.889 | Altered group |
| STAT3_PY705 |  | -0.09 | 0.128 | 0.889 | Unaltered group |
| PECAM1 | 17q23.3 | -0.05 | 0.133 | 0.889 | Unaltered group |
| BAK1 | 6p21.31 | -0.08 | 0.137 | 0.889 | Unaltered group |
| ITGA2 | 5q11.2 | -0.1 | 0.138 | 0.889 | Unaltered group |
| BECN1 | 17q21.31 | -0.06 | 0.143 | 0.889 | Unaltered group |
| MAPK8_PT183_Y185 |  | -0.08 | 0.145 | 0.889 | Unaltered group |
| EIF4EBP1_PT37 |  | 0.12 | 0.145 | 0.889 | Altered group |
| PREX1 | 20q13.13 | -0.1 | 0.151 | 0.889 | Unaltered group |
| CDKN1B | 12p13.1 | -0.09 | 0.152 | 0.889 | Unaltered group |
| PRKAA1 | 5p13.1 | 0.07 | 0.153 | 0.889 | Altered group |
| GSK3A_PS21_S9 |  | 0.09 | 0.157 | 0.889 | Altered group |
| EIF4EBP1_PS65 |  | 0.07 | 0.176 | 0.889 | Altered group |
| XBP1 | 22q12.1 | -0.07 | 0.177 | 0.889 | Unaltered group |
| NRG1 | 8p12 | -0.07 | 0.183 | 0.889 | Unaltered group |
| SRSF1 | 17q22 | -0.04 | 0.185 | 0.889 | Unaltered group |
| GATA3 | 10p14 | -0.05 | 0.185 | 0.889 | Unaltered group |
| EGFR | 7p11.2 | 0.09 | 0.185 | 0.889 | Altered group |
| DIRAS3 | 1p31.3 | -0.09 | 0.194 | 0.889 | Unaltered group |
| FOXO3 | 6q21 | -0.03 | 0.195 | 0.889 | Unaltered group |
| INPP4B | 4q31.21 | -0.13 | 0.201 | 0.889 | Unaltered group |
| CDKN1A | 6p21.2 | -0.08 | 0.219 | 0.889 | Unaltered group |
| CDH3 | 16q22.1 | -0.05 | 0.228 | 0.889 | Unaltered group |
| BRCA2 | 13q13.1 | -0.05 | 0.23 | 0.889 | Unaltered group |
| SMAD4 | 18q21.2 | -0.03 | 0.231 | 0.889 | Unaltered group |
| TP53 | 17p13.1 | -0.1 | 0.233 | 0.889 | Unaltered group |
| YAP1_PS127 |  | 0.14 | 0.243 | 0.889 | Altered group |
| EEF2K | 16p12.2 | 0.09 | 0.246 | 0.889 | Altered group |
| RAD50 | 5q31.1 | -0.05 | 0.246 | 0.889 | Unaltered group |
| DVL3 | 3q27.1 | 0.07 | 0.258 | 0.889 | Altered group |
| MSH6 | 2p16.3 | 0.09 | 0.26 | 0.889 | Altered group |
| PDK1_PS241 |  | 0.06 | 0.262 | 0.889 | Altered group |
| ERBB2_PY1248 |  | -0.05 | 0.262 | 0.889 | Unaltered group |
| GSK3A | 19q13.2 | 0.04 | 0.263 | 0.889 | Altered group |
| GSK3B | 3q13.33 | 0.04 | 0.263 | 0.889 | Altered group |
| ACVRL1 | 12q13.13 | -0.03 | 0.281 | 0.889 | Unaltered group |
| NFKB1_PS536 |  | 0.13 | 0.285 | 0.889 | Altered group |
| ACACA | 17q12 | 0.15 | 0.286 | 0.889 | Altered group |
| MSH2 | 2p21-p16.3 | -0.08 | 0.288 | 0.889 | Unaltered group |
| CCNB1 | 5q13.2 | 0.1 | 0.297 | 0.889 | Altered group |
| AKT1 | 14q32.33 | 0.07 | 0.311 | 0.889 | Altered group |
| AKT2 | 19q13.2 | 0.07 | 0.311 | 0.889 | Altered group |
| AKT3 | 1q43-q44 | 0.07 | 0.311 | 0.889 | Altered group |
| CDH2 | 18q12.1 | -0.04 | 0.313 | 0.889 | Unaltered group |
| BAP1 | 3p21.1 | -0.12 | 0.315 | 0.889 | Unaltered group |
| BAX | 19q13.33 | 0.07 | 0.322 | 0.889 | Altered group |
| PRKCB_PS660 |  | 0.08 | 0.332 | 0.889 | Altered group |
| SYK | 9q22.2 | -0.14 | 0.333 | 0.889 | Unaltered group |
| AKT1_PT308 |  | 0.09 | 0.339 | 0.889 | Altered group |
| AKT2_PT308 |  | 0.09 | 0.339 | 0.889 | Altered group |
| AKT3_PT308 |  | 0.09 | 0.339 | 0.889 | Altered group |
| ARAF_PS299 |  | -0.02 | 0.349 | 0.889 | Unaltered group |
| MRE11 | 11q21 | -0.03 | 0.351 | 0.889 | Unaltered group |
| RICTOR | 5p13.1 | -0.11 | 0.351 | 0.889 | Unaltered group |
| PRKAA1_PT172 |  | 0.08 | 0.355 | 0.889 | Altered group |
| EIF4EBP1_PT70 |  | 0.04 | 0.358 | 0.889 | Altered group |
| EGFR_PY1173 |  | -0.02 | 0.359 | 0.889 | Unaltered group |
| YAP1 | 11q22.1 | 0.04 | 0.365 | 0.889 | Altered group |
| TSC1 | 9q34 | 0.05 | 0.365 | 0.889 | Altered group |
| RPS6KA1 | 1p36.11 | -0.04 | 0.367 | 0.889 | Unaltered group |
| WWTR1 | 3q25.1 | -0.02 | 0.371 | 0.889 | Unaltered group |
| NDRG1_PT346 |  | -0.12 | 0.376 | 0.889 | Unaltered group |
| RAF1_PS338 |  | -0.03 | 0.376 | 0.889 | Unaltered group |
| BAD_PS112 |  | 0.05 | 0.379 | 0.889 | Altered group |
| ESR1 | 6q25.1-q25.2 | -0.16 | 0.381 | 0.889 | Unaltered group |
| BCL2L11 | 2q13 | -0.08 | 0.383 | 0.889 | Unaltered group |
| MTOR_PS2448 |  | 0.03 | 0.401 | 0.889 | Altered group |
| EIF4G1 | 3q27.1 | 0.07 | 0.405 | 0.889 | Altered group |
| G6PD | Xq28 | -0.05 | 0.405 | 0.889 | Unaltered group |
| TFRC | 3q29 | 0.11 | 0.406 | 0.889 | Altered group |
| SRC_PY416 |  | -0.06 | 0.416 | 0.889 | Unaltered group |
| PDCD4 | 10q25.2 | 0.07 | 0.418 | 0.889 | Altered group |
| TUBA1B | 12q13.12 | 0.08 | 0.423 | 0.889 | Altered group |
| YBX1_PS102 |  | 0.03 | 0.424 | 0.889 | Altered group |
| JUN_PS73 |  | -0.04 | 0.429 | 0.889 | Unaltered group |
| PRDX1 | 1p34.1 | -0.05 | 0.433 | 0.889 | Unaltered group |
| RPS6 | 9p22.1 | 0.1 | 0.438 | 0.889 | Altered group |
| MET_PY1235 |  | -0.02 | 0.451 | 0.889 | Unaltered group |
| CTNNB1 | 3p22.1 | 0.1 | 0.453 | 0.889 | Altered group |
| VHL | 3p25.3 | -0.2 | 0.454 | 0.889 | Unaltered group |
| CCND1 | 11q13.3 | -0.02 | 0.462 | 0.889 | Unaltered group |
| CDH1 | 16q22.1 | 0.12 | 0.464 | 0.889 | Altered group |
| RPS6KB1 | 17q23.1 | 0.04 | 0.467 | 0.889 | Altered group |
| FOXO3_PS318_S321 |  | -0.02 | 0.483 | 0.889 | Unaltered group |
| PEA15 | 1q23.2 | 0.05 | 0.487 | 0.889 | Altered group |
| SHC1_PY317 |  | -0.03 | 0.492 | 0.889 | Unaltered group |
| CHEK2 | 22q12.1 | 0.05 | 0.509 | 0.889 | Altered group |
| MAP2K1 | 15q22.31 | 0.03 | 0.512 | 0.889 | Altered group |
| MAPK1 | 22q11.22 | 0.05 | 0.521 | 0.889 | Altered group |
| ETS1 | 11q24.3 | -0.05 | 0.533 | 0.889 | Unaltered group |
| MAPK1_PT202_Y204 |  | -0.09 | 0.534 | 0.889 | Unaltered group |
| MAPK3_PT202_Y204 |  | -0.09 | 0.534 | 0.889 | Unaltered group |
| BIRC2 | 11q22.2 | 0.03 | 0.539 | 0.889 | Altered group |
| TSC2 | 16p13.3 | 0.05 | 0.541 | 0.889 | Altered group |
| MAPK9 | 5q35.3 | 0.04 | 0.542 | 0.889 | Altered group |
| KIT | 4q12 | 0.06 | 0.543 | 0.889 | Altered group |
| ESR1_PS118 |  | 0.03 | 0.544 | 0.889 | Altered group |
| PXN | 12q24.23 | -0.04 | 0.547 | 0.889 | Unaltered group |
| RBM15 | 1p13.3 | 0.05 | 0.548 | 0.889 | Altered group |
| RPS6_PS235_S236 |  | 0.07 | 0.55 | 0.889 | Altered group |
| TGM2 | 20q11.23 | 0.06 | 0.552 | 0.889 | Altered group |
| CHEK1 | 11q24.2 | -0.02 | 0.555 | 0.889 | Unaltered group |
| SERPINE1 | 7q22.1 | -0.11 | 0.555 | 0.889 | Unaltered group |
| NOTCH1 | 9q34.3 | 0.02 | 0.556 | 0.889 | Altered group |
| TSC2_PT1462 |  | -0.04 | 0.557 | 0.889 | Unaltered group |
| XRCC5 | 2q35 | 0.03 | 0.561 | 0.889 | Altered group |
| ATM | 11q22.3 | 0.07 | 0.563 | 0.889 | Altered group |
| BRAF | 7q34 | 0.05 | 0.567 | 0.889 | Altered group |
| SRC_PY527 |  | -0.06 | 0.569 | 0.889 | Unaltered group |
| MYC | 8q24.21 | -0.04 | 0.575 | 0.889 | Unaltered group |
| RAB25 | 1q22 | -0.07 | 0.579 | 0.889 | Unaltered group |
| MAPK14 | 6p21.31 | 0.03 | 0.581 | 0.889 | Altered group |
| EGFR_PY1068 |  | -0.05 | 0.587 | 0.891 | Unaltered group |
| CDKN1B_PT198 |  | -0.01 | 0.604 | 0.91 | Unaltered group |
| EIF4EBP1 | 8p11.23 | 0.03 | 0.623 | 0.913 | Altered group |
| IRS1 | 2q36.3 | 0.03 | 0.624 | 0.913 | Altered group |
| RICTOR_PT1135 |  | -0.02 | 0.626 | 0.913 | Unaltered group |
| AR | Xq12 | 0.06 | 0.628 | 0.913 | Altered group |
| RPS6KB1_PT389 |  | -0.07 | 0.631 | 0.913 | Unaltered group |
| STK11 | 19p13.3 | 0.02 | 0.632 | 0.913 | Altered group |
| CHEK1_PS345 |  | -0.02 | 0.641 | 0.914 | Unaltered group |
| EIF4E | 4q23 | 0.02 | 0.645 | 0.914 | Altered group |
| CDKN1B_PT157 |  | 0.02 | 0.647 | 0.914 | Altered group |
| ERBB3 | 12q13.2 | -0.02 | 0.662 | 0.928 | Unaltered group |
| COL6A1 | 21q22.3 | -0.03 | 0.67 | 0.929 | Unaltered group |
| YBX1 | 1p34.2 | -0.02 | 0.672 | 0.929 | Unaltered group |
| ERBB2 | 17q12 | -0.04 | 0.682 | 0.929 | Unaltered group |
| SQSTM1 | 5q35.3 | -0.07 | 0.685 | 0.929 | Unaltered group |
| RPS6KA1_PT359_S363 |  | -0.02 | 0.7 | 0.929 | Unaltered group |
| FASN | 17q25.3 | -0.08 | 0.709 | 0.929 | Unaltered group |
| GAPDH | 12p13.31 | 0.08 | 0.714 | 0.929 | Altered group |
| PRKCD_PS664 |  | 0.01 | 0.715 | 0.929 | Altered group |
| ERBB3_PY1298 |  | -0.02 | 0.724 | 0.929 | Unaltered group |
| CASP7 | 10q25.3 | 0.05 | 0.73 | 0.929 | Altered group |
| GSK3A_PS9 |  | 0.03 | 0.733 | 0.929 | Altered group |
| GSK3B_PS9 |  | 0.03 | 0.733 | 0.929 | Altered group |
| LCK | 1p35.2 | -0.02 | 0.74 | 0.929 | Unaltered group |
| PEA15_PS116 |  | -0.04 | 0.745 | 0.929 | Unaltered group |
| PTEN | 10q23.31 | 0.02 | 0.759 | 0.929 | Altered group |
| CDK1 | 10q21.2 | 0.01 | 0.76 | 0.929 | Altered group |
| RB1_PS807_S811 |  | -0.03 | 0.762 | 0.929 | Unaltered group |
| MAPK14_PT180_Y182 |  | 0.03 | 0.764 | 0.929 | Altered group |
| PCNA | 20p12.3 | 0.01 | 0.769 | 0.929 | Altered group |
| FOXM1 | 12p13.33 | -0.03 | 0.772 | 0.929 | Unaltered group |
| DIABLO | 12q24.31 | -0.03 | 0.779 | 0.929 | Unaltered group |
| STMN1 | 1p36.11 | -0.01 | 0.779 | 0.929 | Unaltered group |
| TP53BP1 | 15q15.3 | 0.02 | 0.781 | 0.929 | Altered group |
| MS4A1 | 11q12.2 | -0.02 | 0.783 | 0.929 | Unaltered group |
| RAD51 | 15q15.1 | -0.01 | 0.784 | 0.929 | Unaltered group |
| PIK3CA | 3q26.32 | -0.01 | 0.793 | 0.929 | Unaltered group |
| MAP2K1_PS217_S221 |  | 0.02 | 0.801 | 0.929 | Altered group |
| FN1 | 2q35 | 0.03 | 0.809 | 0.929 | Altered group |
| XRCC1 | 19q13.31 | 0.01 | 0.811 | 0.929 | Altered group |
| KDR | 4q12 | -0.02 | 0.82 | 0.929 | Unaltered group |
| RAB11A | 15q22.31 | -0.01 | 0.827 | 0.929 | Unaltered group |
| RAB11B | 19p13.2 | -0.01 | 0.827 | 0.929 | Unaltered group |
| PIK3R1 | 5q13.1 | -0.01 | 0.833 | 0.929 | Unaltered group |
| PIK3R2 | 19p13.11 | -0.01 | 0.833 | 0.929 | Unaltered group |
| STAT5A | 17q21.2 | 0.02 | 0.835 | 0.929 | Altered group |
| RPS6_PS240_S244 |  | 0.03 | 0.836 | 0.929 | Altered group |
| BCL2L1 | 20q11.21 | -0.01 | 0.837 | 0.929 | Unaltered group |
| SMAD3 | 15q22.33 | 0.01 | 0.841 | 0.929 | Altered group |
| CAV1 | 7q31.2 | 0.03 | 0.842 | 0.929 | Altered group |
| SRC | 20q11.23 | 0.01 | 0.874 | 0.96 | Altered group |
| BID | 22q11.21 | 0.01 | 0.885 | 0.966 | Altered group |
| PARK7 | 1p36.23 | 0.01 | 0.895 | 0.973 | Altered group |
| CHEK2_PT68 |  | 0 | 0.91 | 0.974 | Altered group |
| CCNE1 | 19q12 | -0.02 | 0.913 | 0.974 | Unaltered group |
| EEF2 | 19p13.3 | 0.01 | 0.914 | 0.974 | Altered group |
| ACACA_PS79 |  | 0.01 | 0.921 | 0.974 | Altered group |
| ACACB_PS79 |  | 0.01 | 0.921 | 0.974 | Altered group |
| YWHAZ | 8q22.3 | 0.01 | 0.932 | 0.98 | Altered group |
| PDK1 | 2q31.1 | 0 | 0.94 | 0.98 | Altered group |
| RPTOR | 17q25.3 | 0 | 0.941 | 0.98 | Altered group |
| AKT1_PS473 |  | 0.01 | 0.97 | 0.991 | Altered group |
| AKT2_PS473 |  | 0.01 | 0.97 | 0.991 | Altered group |
| AKT3_PS473 |  | 0.01 | 0.97 | 0.991 | Altered group |
| GAB2 | 11q14.1 | 0 | 0.975 | 0.991 | Unaltered group |
| CLDN7 | 17p13.1 | 0 | 0.977 | 0.991 | Altered group |
| ERCC1 | 19q13.32 | 0 | 0.981 | 0.991 | Unaltered group |
| NF2 | 22q12.2 | 0 | 0.997 | 0.997 | Altered group |
| AKT1S1_PT246 |  | 0 | 0.997 | 0.997 | Altered group |
